# Assessment of MRI parameters for studying brain development in newborns with congenital heart disease

**DOI:** 10.1186/1532-429X-17-S1-P205

**Published:** 2015-02-03

**Authors:** Brahmdeep S Saini, Prakash Muthusami, Sujana Madathil, Jessie Mei Lim, Christopher Macgowan, Steven Miller, Mike Seed

**Affiliations:** Diagnostic Imaging, The Hospital for Sick Children, Toronto, ON Canada; Heart Centre, The Hospital for Sick Children, Toronto, ON Canada; Physiology and Experimental Medicine, The Hospital for Sick Children, Toronto, ON Canada; Neurology, The Hospital for Sick Children, Toronto, ON Canada

## Background

Microstructural evidence of white matter (WM) dysmaturation in the brains of newborns with congenital heart disease (CHD) using MRI has been previously shown [1]. Further, delayed WM development confers an increased risk of WM injury before and after neonatal cardiac surgery [2]. WM injury is associated with a high incidence of subsequent neurodevelopmental deficits and fetal interventions to improve brain development in the setting of CHD are currently being investigated. We wished to compare the utility of our previously described parameters in another cohort of CHD newborns, and assess the performance of a new parameter (WM T2 relaxation) for discerning differences in WM maturation.

## Methods

We studied the newborn brains of 30 normal and 21 CHD subjects between June 2013 and September 2014 as part of a hospital IRB approved study. MRI was performed without sedation at a mean age of 7 days (range 0-42 days) on a Siemens Avanto 1.5T system (Erlangen) with the following sequences: high resolution 3D T2W FSE, multivoxel proton magnetic resonance spectroscopy and diffusion tensor imaging. T2 mapping was performed in 6 normal and 6 CHD newborns. We calculated brain volume by segmenting the 3D T2W images using Mimics (Materialise, Leuven). The N-acetyl acetate to choline (NAA/Chol) ratio was calculated from the MRS of the centrum semiovale. Regions of interest for analysis of T2, fractional anisotropy (FA) and apparent diffusion coefficient (ADC) included inferior frontal, superior frontal and parietal WM. An unpaired t-test was used to determine the statistical significance of differences between the two groups.

## Results

**Figure 1:**
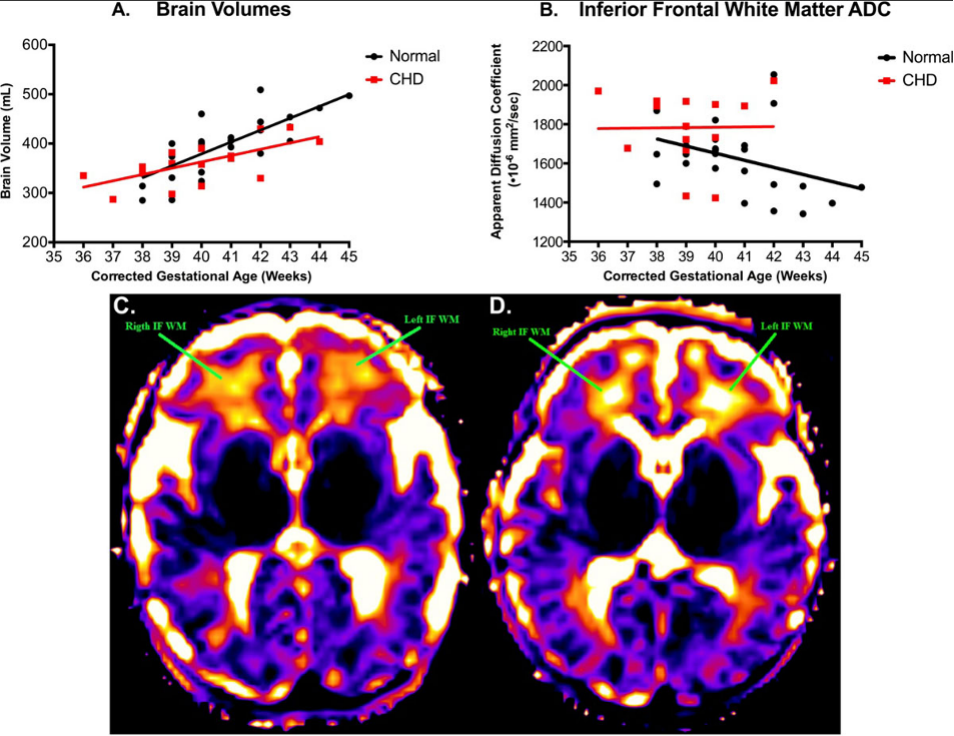
Comparison of brain volumes (A.) and inferior frontal WM ADC (B.) versus corrected gestational age (GA) between CHD newborns and controls. Visual comparison of brain T2 maps at the same window level of inferior frontal WM in a control (C.) to a CHD newborn (D.). Both the normal and CHD newborn were of the same age, 39 weeks corrected GA.

There was no significant difference between the corrected gestational ages of the two groups (p=0.88). Brain volume increased with age but was lower in CHD newborns than controls (Fig. 1A). The ADC values decreased with age but were higher in CHD newborns than in controls (Fig. 1B). FA and NAA/Chol ratios both increased with age but were not significantly different between the two groups. T2s decreased with age and the average WM T2s of CHD newborns were higher than controls. The T2 brain maps of CHD newborns showed visual differences in comparison to controls of similar age (Fig. 1C-D). Table 1 summarizes all the results.

**Table 1:** Comparison of MRI parameters in assessing brain development between normal and CHD newborns.

MRI Parameter	Control Mean ± SD	CHD Mean ± SD	P Value	Significant (Y/N)
**Brain Volume (mL)**	392 ± 57, n=29	362 ± 41, n=18	0.04	Y
**MRS:** **Centrum Semiovale NAA/Chol Ratio**	0.63 ± 0.14, n=28	0.67 ± 0.11, n=18	0.28	N
**WM T2 (ms)**	269 ± 27, n=6	293 ± 39, n=6	0.04	Y
**Fractional Anisotropy (x10** ^**-3**^ **):** **Inferior Frontal WM** **Superior Frontal WM** **Parietal WM**	162 ± 42, n=27 177 ± 39, n=29 182 ± 45, n=29	165 ± 73, n=14 185 ± 66, n=15 183 ± 70, n=15	0.89 0.60 0.98	N N N
**Apparent Diffusion Coefficient (x10** ^**-6**^ **mm** ^**2**^ **/sec):** **Inferior Frontal WM** **Superior Frontal WM** **Parietal WM**	1632 ± 171, n=27 1504 ± 162, n=29 1691 ± 170, n=29	1783 ± 186, n=14 1552 ± 172, n=15 1818 ± 181, n=15	0.01 0.37 0.03	Y N Y

MRS - magnetic resonance spectroscopy, WM - white matter.

## Conclusions

As expected, WM ADC values in CHD newborns were significantly higher than controls. We also found a reduction in brain volume in newborns with CHD, similar to the results of other groups [3]. In unmyelinated WM regions, FA and NAA/Chol ratios were not significantly different. Whereas, WM T2 was significantly higher in CHD newborns, despite the smaller number of studies that incorporated T2 mapping. WM T2 may be a sensitive marker of WM dysmaturation in the setting of CHD and a useful adjunct to more established parameters in the assessment of the impact of fetal interventions on brain development.
